# RAPTOR up-regulation contributes to resistance of renal cancer cells to PI3K-mTOR inhibition

**DOI:** 10.1371/journal.pone.0191890

**Published:** 2018-02-01

**Authors:** Philip Earwaker, Caroline Anderson, Frances Willenbrock, Adrian L. Harris, Andrew S. Protheroe, Valentine M. Macaulay

**Affiliations:** 1 Department of Oncology, Oxford, United Kingdom; 2 Oxford Cancer and Haematology Centre, Oxford University Hospitals NHS Trust, Churchill Hospital, Oxford, United Kingdom; Univerzitet u Beogradu, SERBIA

## Abstract

The outlook for patients with advanced renal cell cancer (RCC) has been improved by targeted agents including inhibitors of the PI3 kinase (PI3K)-AKT-mTOR axis, although treatment resistance is a major problem. Here, we aimed to understand how RCC cells acquire resistance to PI3K-mTOR inhibition. We used the RCC4 cell line to generate a model of *in vitro* resistance by continuous culture in PI3K-mTOR kinase inhibitor NVP-BEZ235 (BEZ235, Dactolisib). Resistant cells were cross-resistant to mTOR inhibitor AZD2014. Sensitivity was regained after 4 months drug withdrawal, and resistance was partially suppressed by HDAC inhibition, supporting an epigenetic mechanism. BEZ235-resistant cells up-regulated and/or activated numerous proteins including MET, ABL, Notch, IGF-1R, INSR and MEK/ERK. However, resistance was not reversed by inhibiting or depleting these pathways, suggesting that many induced changes were passengers not drivers of resistance. BEZ235 blocked phosphorylation of mTOR targets S6 and 4E-BP1 in parental cells, but 4E-BP1 remained phosphorylated in resistant cells, suggesting BEZ235-refractory mTORC1 activity. Consistent with this, resistant cells over-expressed mTORC1 component RAPTOR at the mRNA and protein level. Furthermore, BEZ235 resistance was suppressed by RAPTOR depletion, or allosteric mTORC1 inhibitor rapamycin. These data reveal that RAPTOR up-regulation contributes to PI3K-mTOR inhibitor resistance, and suggest that RAPTOR expression should be included in the pharmacodynamic assessment of mTOR kinase inhibitor trials.

## Introduction

Treatment of metastatic renal cell cancer (RCC) has been transformed by introduction of targeted agents, including multi-targeted inhibitors of VEGF receptor and other tyrosine kinases, and inhibitors of the mammalian target of rapamycin (mTOR) [1]. mTOR is a serine threonine kinase that exists in two protein complexes: mTOR complex 1 (mTORC1) and 2 (mTORC2) [2]. The principal function of mTORC1 is to promote translation, by phosphorylating two key substrates. First, mTORC1-dependent phosphorylation of S6 kinase (S6K) allows S6K to phosphorylate its target S6 ribosomal peptide, often used as a measure of mTOR activity [3]. Secondly, phosphorylation of the eukaryotic initiation factor 4E binding protein 1 (4E-BP1) results in dissociation of 4E-BP1 from eukaryotic initiation of translation factor 4E (eIF4E), which is then able to enter the eIF4F complex to initiate cap-dependent translation [4]. Thus mTORC1 promotes synthesis of proteins required for cell growth and proliferation, while mTORC2 is required for phosphorylation of S473 AKT leading to mTORC1 activation, cytoskeletal organisation, cell survival and metabolism [5–7].

The mTOR inhibitors licensed for clinical use are rapalogs temsirolimus and everolimus, both derived from the parent molecule rapamycin [8]. These are allosteric mTOR inhibitors that bind the intracellular FK506-binding protein FKBP12; this complex interacts with mTOR at a site distant from the kinase domain, causing mTOR to dissociate from the unique mTORC1 component Regulatory-Associated Protein of mTOR complex 1 (RAPTOR) [2, 9]. Rapalogs have relatively modest clinical activity [10, 11], prompting development of inhibitors of mTOR kinase that inhibit both mTORC1 and mTORC2, including AZD8055, AZD2014 and PP242 [12–14]. Many mTOR kinase inhibitors also inhibit the closely related PI3K, and a number of these agents have undergone early phase clinical testing, including NVP-BEZ235 (BEZ235, Dactolisib), PF-05212384, GDC-0980 (apitolisib) and BGT226 [15–19].

It is clear that although there are now numerous targeted therapies in development for treatment of RCC, response rates are low, and time to progression remains short [1]. Primary and acquired resistance to these drugs is a real clinical problem; it is important to understand the basis of resistance, in order to identify biomarkers for patient selection, and identify combination treatments that may overcome resistance. Here, we used RCC cells to generate a model of induced resistance to the dual PI3K-mTOR kinase inhibitor BEZ235. BEZ235 is a potent inhibitor of Class I PI3Ks with IC_50_ values of 4, 75 and 7 nM for inhibition of p110α, p110β and p110δ respectively, and 6.5 nM for inhibition of mTOR kinase [20]. We showed that resistance was reversed on prolonged drug-free culture, consistent with a non-genomic resistance mechanism. Compared with BEZ235-sensitive parental cells, the resistant subline exhibited changes in expression and activation states of numerous proteins and pathways, but only one was shown to contribute to resistance. This was BEZ235-refractory activation of mTORC1, manifest as persistent phosphorylation of 4E-BP1, associated with RAPTOR up-regulation. Phosphorylation of 4E-BP1 was suppressed, and BEZ235 resistance partially reversed, by RAPTOR knockdown or mTORC1 inhibition using rapamycin. These data identify RAPTOR as a novel mediator of resistance to mTOR kinase inhibition in renal cancer.

## Results

### RCC cells induced to be resistant to PI3K-mTOR inhibitor BEZ235 are cross-resistant to AZD2014

**Fig 1:**
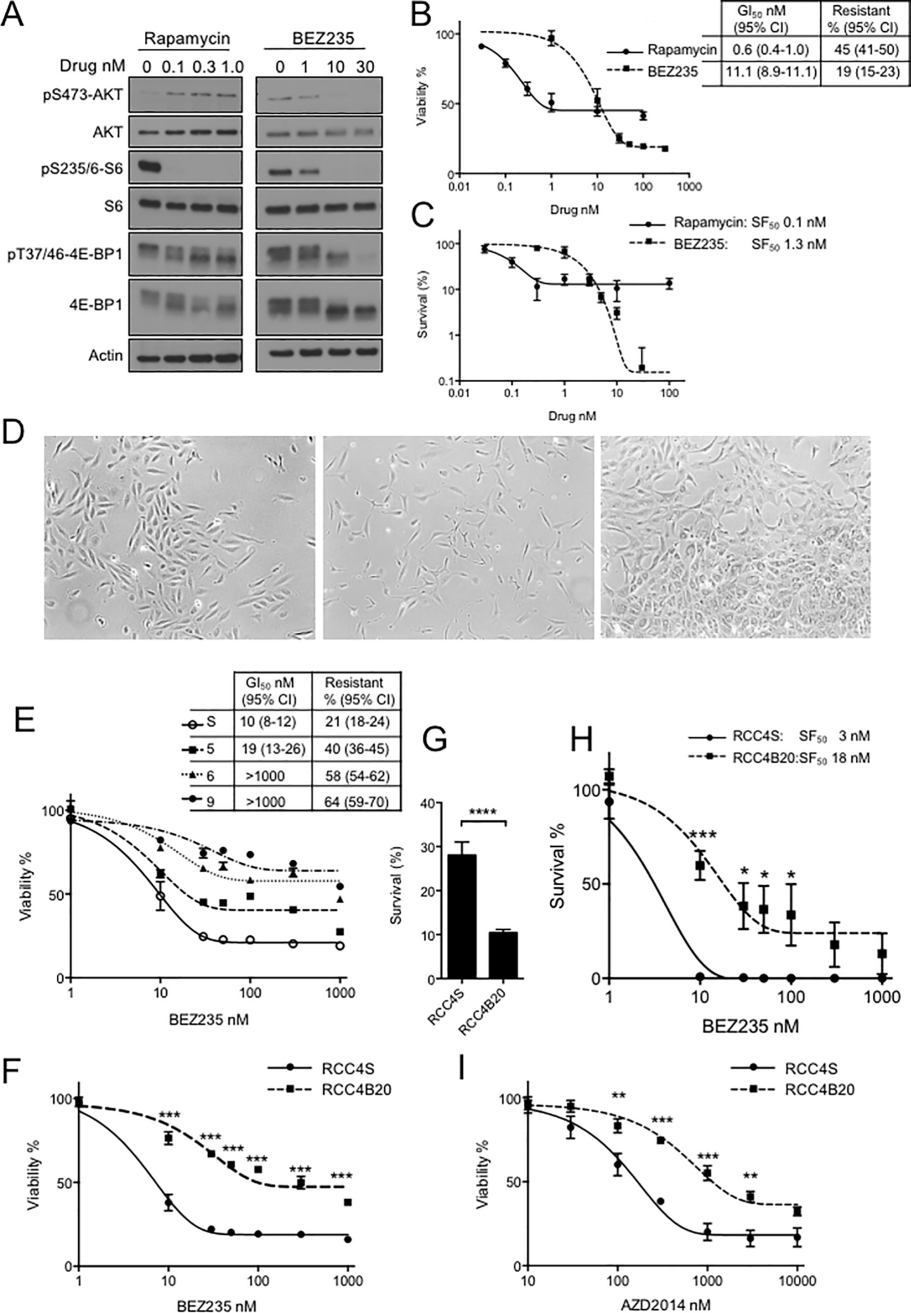
BEZ235-resistant RCC cells are cross-resistant to AZD2014. **A.** RCC4 EV cells were treated with rapamycin, BEZ235 or solvent (control) for 6 hr, cells were lysed and analysed by western blot. **B.** Response of RCC4 EV cells to rapamycin or BEZ235 was tested by viability assay. Graph: mean ± SEM of three independent experiments, showing GI_50_ values and resistant populations (% cells remaining viable at the plateau of the dose-response curve). C. Clonogenic survival assays to assess response of RCC4 EV cells to rapamycin or BEZ235. Graphs: mean ± SEM of three independent experiments, with mean SF_50_ values. **D.** Light micrographs, original magnification x4, of left: parental (BEZ235-sensitive) RCC4 cells; center: RCC4 cells after one week of exposure to 20 nM BEZ235, showing spindle-like morphology; right: border of a dense colony of BEZ235-resistant cells (bottom right) emerging after 3 months exposure to BEZ235, on a background of sparse cells (top left). **E.** Serial viability assays performed after 5–9 weeks after RCC4 cells underwent morphological change, compared with RCC4 cells cultured in solvent containing medium for the same duration (RCC4S). Graph: mean ± SEM viability, showing in legend the BEZ235 GI_50_ values and resistant populations. **F.** Effect of BEZ235 on mean ± SEM viability of RCC4S and resistant RCC4B20 cells, after attainment of stable resistance 3 months post morphological change. Dose-response curves were significantly different by 2-way ANOVA (***p<0.001). Table 2 shows the derived BEZ235 GI_50_ concentrations and resistant populations, and fold change calculated as ratio of these parameters in resistant/sensitive cells. **G.** Survival of RCC4S and RCC4B20 cells in the absence of BEZ235, expressed as mean ± SEM % cells seeded (****p<0.0001 by t-test). In subsequent survival assays, 3000 RCC4S and 6000 RCC4B20 cells were seeded per 10 cm dish, to give similar numbers of surviving colonies in BEZ235-untreated controls. **H.** Effect of BEZ235 on cell survival. Graph: mean ± SEM of 3 independent experiments (*p<0.05, ***p<0.001). **I.** Effect of AZD2014 on mean ± SEM viability, showing that RCC4B20 cells were significantly more resistant to AZD2014 than RCC4S cells (**p<0.01, ***p<0.001).

We used RCC4-EV cells that harbor inactivating VHL mutation, often found in clear cell RCC (CCRCC) [21] to generate a model of induced resistance to PI3K-mTOR kinase inhibitor BEZ235 [20]. Initial experiments compared the ability of BEZ235 and rapamycin to inhibit cell signalling and viability. Phosphorylation of S473 AKT was enhanced by rapamycin and blocked by BEZ235. Both drugs inhibited mTORC1-induced phosphorylation of S6 and 4EBP1, the latter suppressed more potently by BEZ235 (Fig 1A). Both agents also inhibited cell viability, with lower GI_50_ values in cells treated with rapamycin, although the resistant population, assessed as % viability at the dose-response plateau at >100nM drug, was lower in BEZ235-treated cells (Fig 1B). A similar pattern of relative efficacy was apparent in survival assays (Fig 1C). We noted that inhibition of 4E-BP1 phosphorylation (Fig 1A) tracked with the ability to block viability and cell survival, while phospho-AKT and phospho-S6 did not. Indeed, others have reported that BEZ235-induced inhibition of 4E-BP1 phosphorylation correlates with inhibition of proliferation [22, 23].

**Fig 2:**
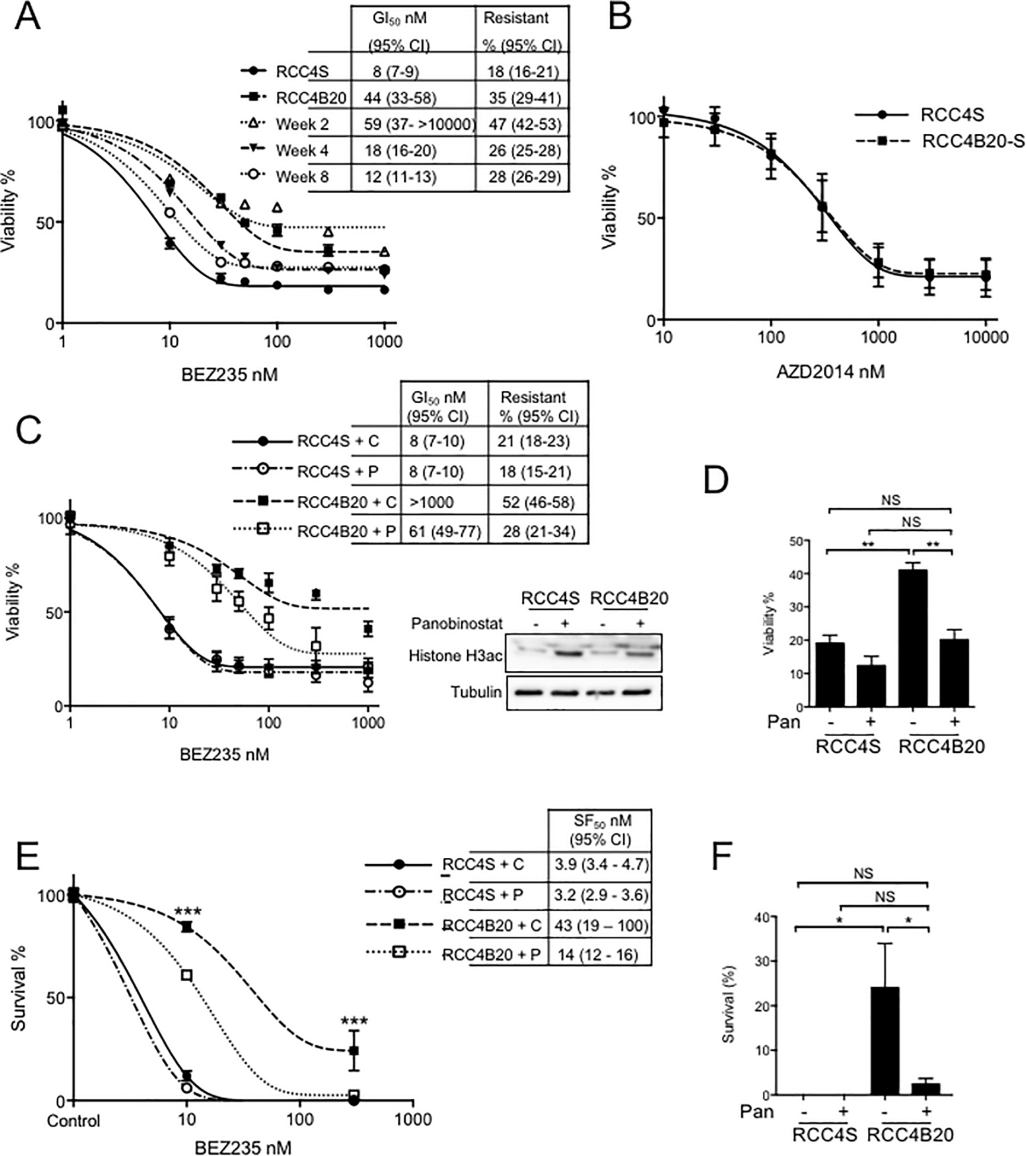
Induced BEZ235 resistance is reversible, and antagonized by HDAC inhibition. **A.** BEZ235-response of RCC4B20 cells after 2–8 weeks culture without BEZ235, showing relative viability compared with RCC4S cells, and RCC4B20 maintained in drug. Legend gives GI_50_ values and resistant populations. **B.** Effect of AZD2014 on viability of RCC4S and re-sensitized RCC4B20 cells (RCC4B20-S), after >3 months in BEZ235-free medium. **C.** Cells were treated with BEZ235 with solvent control (C) or 20 nM Panobinostat (P). Panobinostat. Graph shows mean ± SEM relative viability, with GI_50_ values and % resistant populations. Panobinostat had no effect on RCC4S but partially re-sensitized RCC4B20 cells (>16 fold reduction in BEZ235 GI_50_ value). Blot to right: cells were treated with 20 nM Panobinostat for 1 hr and cell lysates analysed by western blotting for acetylated Histone H3. **D.** Bar graph: relative viability at 1000nM BEZ235. Viability in panobinostat (Pan)-treated RCC4B20 cells was significantly reduced compared with solvent-treated RCC4B20 controls (**p<0.01 by one way ANOVA), and not significantly different from viability in RCC4S cells. **E.** Survival assay of RCC4S and RCC4B20 cells treated as E, with SF_50_ values. Panobinostat did not affect survival of RCC4S cells, but significantly reduced survival of RCC4B20 cells (***p<0.001). **F.** Bar graph: relative cell survival at 300nM BEZ235 (*p<0.05).

Having characterized acute effects of mTOR inhibition, we then established a resistance model by long-term culture in BEZ235. RCC4-EV cells were cultured initially in 10 nM and after recovery of proliferation rate, in 20 nM BEZ235. Further increase in BEZ235 concentration was not tolerated *in vitro* in RCC4 cells. After three months in 20 nM BEZ235, the cells had not grown sufficiently to require passaging, but we noted appearance of dense colonies that rapidly overtook the culture (Fig 1D). From this point, we performed serial viability assays to compare the BEZ235 response of sensitive vehicle-treated parental cells, termed RCC4S, and the resistant subline, RCC4B20 (Fig 1E). BEZ235 resistance progressively increased to a plateau after 3 months, and was quantified by two measures. Firstly, BEZ235 GI_50_ was 14-fold higher in RCC4B20 cells than RCC4S. Secondly there was an increase in the residual resistant population, again calculated as % survival at the plateau of the dose-response curve, at >100nM: ~20% in RCC4S and ~50% in RCC4B20 (Fig 2F; Table 1). RCC4B20 cells were less clonogenic than parental RCC4S cells (Fig 1G), suggesting that they were depleted for stem-like properties. Indeed, we note that BEZ235 is reported to reduce stemness in colon cancer models [24]. However, in terms of relative cell survival upon BEZ235 treatment, the RCC4B20 subline was clearly more resistant, with six-fold increase in BEZ235 SF_50_ (Fig 1H).

**Table 1:** Response of parental and BEZ235-resistant RCC cells to BEZ235 and AZD2014.

	BEZ235		AZD2014	
	GI_50_ nM	Fold change	GI_50_ nM	Fold change
RCC4S	7 (6–8)	14 (8 - >10000)	166 (129–216)	6.8 (4.0–11.4)
RCC4B20	99 (66 - >10000)		1131 (856–1468)	
	BEZ235		AZD2014	
	Resistant (%)	Fold change	Resistant (%)	Fold change
RCC4S	19 (16–21)	2.5 (2–3.3)	18 (13–24)	2 (1.3–3.2)
RCC4B20	47 (42–53)		36 (31–42)	

Table shows GI_50_ concentrations (nM) and % resistant population (% survival at plateau of dose-response curve) expressed as mean (95% confidence intervals) in sensitive RCC4S and resistant RCC4B20 cells interpolated from graphs testing response to BEZ235 (Fig 1F) and AZD2014 (Fig 1I). Fold sensitisation: ratio (95% confidence intervals) of GI_50_ values and resistant populations from three independent experiments using each drug.

Next, RCC4B20 cells were assessed for cross-resistance to alternative PI3K-AKT-mTOR inhibitors, to determine whether the resistance was driven by altered response to PI3K, mTORC1 or mTORC kinase inhibition, and whether relevant to other anti-cancer agents in development. The mTOR kinase inhibitor AZD2014 inhibits mTOR with IC_50_ 2.8 nM and lesser activity against PI3K, with IC_50_ values for α, β, γ, δ, isoforms of p110 of 3.8, >30, >30 and >29 μM respectively [25]. RCC4B20 cells were cross-resistant to AZD2014, with 6.8-fold increase in GI_50_, and 2-fold increase in the resistant population compared with RCC4S cells (Fig 1I, Table 1). In contrast, RCC4B20 cells showed no or trivial cross-resistance to rapamycin, PI3K inhibitor BKM120 or AKT inhibitor AZD5363 (Figure A a-c in S1 File). These results suggest that BEZ235 resistance resulted principally from resistance to mTOR kinase inhibition. Lack of cross-resistance to PI3K-AKT inhibition may be related to the fact that resistant cells could be adapted to BEZ235 concentrations no higher than 20 nM, which is below the BEZ235 IC_50_ value reported for p110β of 75 nM, and above the IC_50_ value of 6.5 nM for inhibition of mTOR kinase [15].

### BEZ235 resistance is reversible on drug removal, and partially suppressed by HDAC inhibition

Induced drug resistance can result from irreversible mutations, or reversible changes mediated at the level of epigenetic regulation [26, 27]. To ascertain whether the induced resistance to BEZ235 was reversible, we tested resistance after drug withdrawal. Over the subsequent 8 weeks BEZ235 resistance was progressively lost, and the re-sensitized cells, RCC4B20-S, also lost cross-resistance to AZD2014 (Fig 2A and 2B). In order to test the contribution of epigenetic changes to this reversible phenotype, we treated RCC cells with panobinostat. This is a potent inhibitor of class I, II and IV histone deacetylases (HDACs), although without detectable clinical activity in patients with refractory RCC [28]. Panobinostat alone caused comparable dose-dependent inhibition of cell viability in RCC4S and RCC4B20 cells (Figure A panel d in S1 File). Panobinostat enhanced Histone H3 acetylation in both parental and BEZ235-resistant cells, confirming its biological activity, and had no effect on the response to BEZ235 in sensitive RCC4S cells (Fig 2C). In contrast, panobinostat sensitized RCC4B20 cells to BEZ235, with reduction in BEZ235 GI_50_ from >1000nM in controls to ~60nM in panobinostat-treated RCC4B20 cells (>16-fold sensitisation; Fig 2C). At 1000nM BEZ235, the viability of Panobinostat-treated RCC4B20 cells was comparable to that of RCC4S cells (Fig 2D). A similar pattern was apparent in clonogenic assays: in the presence of 300nM BEZ235, the survival of Panobinostat-treated RCC4B20 cells was not significantly different from that of parental RCC4S cells (Fig 2E and 2F).

### Dysregulation of multiple signalling pathways in BEZ235-resistant RCC cells

Aiming to identify mediators of BEZ235 resistance, we used three approaches to screen for differences between RCC4S and BEZ235-resistant RCC4B20 cells [29]. Firstly, recognizing the importance of mTOR in regulating signal transduction and translation [2], we investigated changes at the protein level using the Phospho Explorer Array (Full Moon Biosystems). This includes 1318 antibodies to phosphorylated forms of proteins involved in multiple pathways, and the equivalent non-phosphorylated epitopes (see Tables A and B in S2 File). Thirteen proteins or phosphorylations were associated with ≥2-fold change between resistant and sensitive cells (Table 2). We excluded from further analysis hits in which differences between resistant and sensitive cells were also induced by acute BEZ235 treatment of sensitive RCC4S cells (eg phospho-S468 p65 NFκB, Table 2). Nine screen hits underwent low throughput validation by western blot, prioritising actionable changes in three kinase pathways. We were unable to detect phosphorylation on Y1356 MET, the phospho-epitope highlighted in the screen, but in resistant RCC4B20 cells only we detected phosphorylation of Y1234/1235 MET. Total MET was also up-regulated in RCC4B20, with trends to up-regulation of ABL and Mitogen activated protein (MAP) kinase kinase (MEK), and MEK phosphorylation (Figure B a-b in S1 File). Although not a screen hit, MEK target ERK showed up-regulated expression with a trend to increased activation in RCC4B20 cells (Figure B b in S1 File). Six array hits could not be validated (Table 2), presumably reflecting differences in the antibodies and denaturing conditions used here for western blotting, compared with the antibodies and non-denaturing conditions of the array screen. We next assessed whether the three validated hits MET, ABL and MEK-ERK contributed to BEZ235 resistance. The role of MET was tested using MET siRNA and INC280, which inhibits MET trans-phosphorylation [30] (Figure B c-d in S1 File). We also used imatinib [31], confirming inhibition of ABL-induced Y221 phosphorylation of CT10 Regulator of Kinase (CrK) [32], and allosteric MEK inhibitor AZD6244 (selumetinib) [33], which caused sustained ERK inhibition while enhancing MEK phosphorylation at 72hr, presumably a feedback response to allosteric inhibition (Figure B e-f in S1 File). Thus, we confirmed that these agents were capable of depleting or inhibiting their intended targets for the 72hr duration of viability assays (Figure B c-f in S1 File). However, we found no evidence that depleting or inhibiting MET, or inhibiting ABL or MEK-ERK reversed the BEZ235 resistance of RCC4B20 cells (Figure C a-d in S1 File).

**Table 2:** Hits from antibody array.

Protein	Fold Change			Validated
	Resistant:Sensitive	Resistant:Acute	Acute:Sensitive	
Abl1	2.67	2.24	1.19	Y
CaMKII	2.81	1.93	1.46	N
Capase 9	1.6	2.41	0.66	nt
CD4	1.47	2.16	0.68	nt
Integrin beta-1	2.05	1.6	1.28	nt
HSL	1.58	2.36	0.67	N
LIMK1/2	0.7	0.33	2.12	N
MAPKAPK2	0.66	0.5	1.33	N
MEK1	1.5	2.15	0.7	Y
MET pY1356	2.32	1.39	1.67	Y*
NFκB-p65 pS468	4.48	1.37	3.26	N
Ras-GRF1	2.91	1.79	1.56	N
Shc	2.53	1.79	1.41	nt

Table lists screen hits from the Full Moon Biosystems Phospho Explorer antibody array. Hits were those found to have ≥2-fold increase or ≤50% reduction in expression or phosphorylation in RCC4B20 cells compared with RCC4S control treated cells (Resistant:Sensitive) or RCC4B20 cells compared with RCC4S cells acutely-treated with BEZ235 (Resistant:Acute). Validation by western blot was performed in 9 screen hits of which 3 were confirmed to be dysregulated (Y, yes; N, no; nt, not tested).

*Detected increase was in phospho-Y1234/1235 MET (Figure B panel a in S1 File).

In a second approach, we assessed two additional pathways that were not identified as screen hits, but have been associated with response to mTOR inhibition. Type 1 IGF receptor (IGF-1R) is a well-known regulator of PI3K-AKT-mTOR activation [34], and the PI3K-AKT pathway is subject to negative regulation mediated by S6K-induced serine phosphorylation of the IGF-1R docking protein insulin receptor substrate-1 (IRS-1) [35]. Indeed, we previously showed that this effect operates in RCC, and is responsible for AKT activation when this feedback loop is blocked by mTOR inhibition [36]. Here, we did find evidence of IGF-1R and insulin receptor (INSR) up-regulation in RCC4B20 cells compared with controls (Figure B g in S1 File). We used IGF-1R/INSR kinase inhibitor OSI-906 (linsitinib) to block this pathway, but found no evidence that this intervention sensitized RCC4B20 cells to BEZ235 (Figure B h and Figure C e in S1 File), suggesting that IGF-1R/INSR up-regulation was not a driver of resistance. We also tested for dysregulation of the Notch pathway, which had not been represented on the antibody array. On binding of Delta-like and Jagged ligands, Notch receptors undergo regulated cleavage at V1744 by gamma secretase, releasing Notch intracellular domain (NICD), which promotes transcription of Notch target genes [37]. Of relevance to our RCC BEZ235-resistance model, Notch and Jagged 1 are up-regulated in RCC, and Notch inhibition suppresses RCC growth *in vitro* and *in vivo* [38]. Compared with RCC4S cells, BEZ235-resistant RCC4B20 cells contained more Jagged protein and NICD cleaved at V1744, accompanied by up-regulation of Notch target genes *HEY1* and *HES* (Figure B i-k in S1 File). These data indicate Notch pathway activation in BEZ235-resistant cells. The functional consequences of this change were assessed using gamma secretase inhibitor dibenzazepine (DBZ), which after 24hr was confirmed to prevent NICD release in RCC4S and RCC4B20 cells, associated with down-regulation of Notch target HES (Figure B panel k in S1 File). Therefore, to test the contribution of Notch activation to BEZ235 resistance, RCC4 cells were treated with DBZ, and 24hr later with BEZ235. Despite testing under conditions that blocked Notch signalling, DBZ had no effect on the response to BEZ235 (Figure C f in S1 File). Thus, we concluded that activation or overexpression of MET, ABL, MEK-ERK, IGF-1R and Notch are correlates but not mediators of BEZ235 resistance in this RCC model.

### BEZ235 resistant cells show persistent 4EBP1 phosphorylation and RAPTOR upregulation

**Fig 3:**
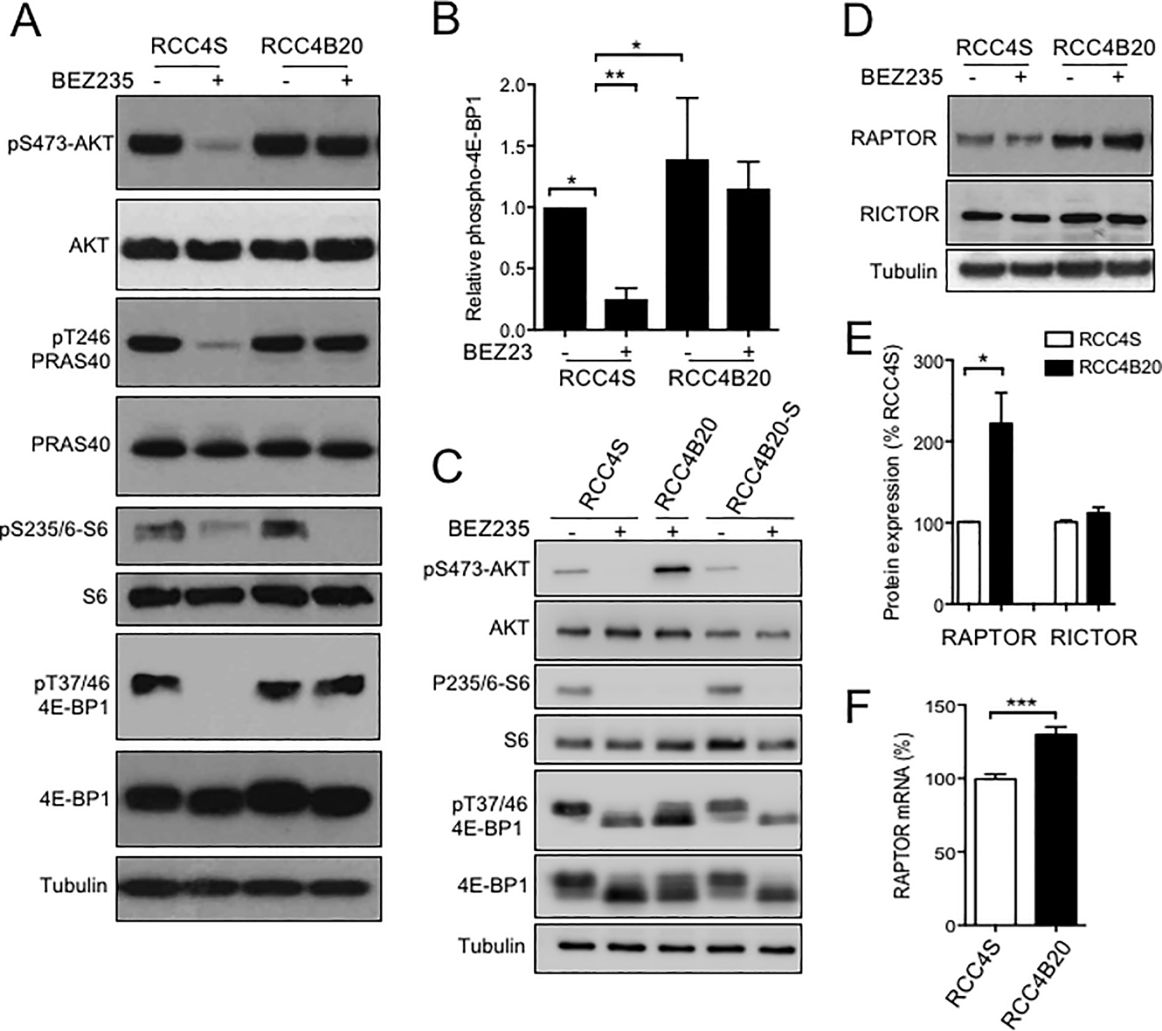
BEZ235-resistant cells exhibit BEZ235-refractory mTORC1 activation. **A.** RCC4S and RCC4B20 cells were treated with solvent or 20 nM BEZ235 for 1 hr, and lysates were analysed by western blot. **B.** Phospho-4E-BP1 signal was quantified from n = 3 independent blots as A), corrected for total 4E-BP1 and expressed as % signal in BEZ235-untreated RCC4S cells (*p<0.05, **p<0.01). **C.** RCC4S and RCC4B20-S cells were treated with solvent or 20 nM BEZ235 for 1 hr, and RCC4B20 cells were treated with fresh 20 nM BEZ235 for 1 hour with no drug washout prior to lysis and western blot as A). **D**. Cells were treated with solvent or 20 nM BEZ235 for 1 hr and lysates analyzed by western blot for RAPTOR and RICTOR. **E.** RAPTOR and RICTOR were quantified from n = 3 western blots as D), corrected for tubulin loading and expressed as mean ± SEM % levels in RCC4S cells (*p<0.05). **F.** Mean ± SEM RAPTOR mRNA quantified by qRT-PCR in RCC4S and RCC4B20 cells (n = 5), corrected for actin, expressed as % expression in RCC4S (***p<0.001).

The array screen had not indicated significant changes in mTOR activity (Table 2, Table A, Table B in S2 File), but as a third approach to investigate BEZ235 resistance, we checked for alterations in this pathway. We found that BEZ235 inhibited S6 phosphorylation in both RCC4S and RCC4B20 cells, providing evidence of mTORC1 inhibition (Fig 3A). This confirmed that BEZ235 was active in the resistant cells, and suggests that drug export was an unlikely contributor to resistance. Acute treatment with BEZ235 also inhibited phosphorylation of 4E-BP1 in RCC4S cells. In contrast, there was persistent BEZ235-resistant 4E-BP1 phosphorylation in RCC4B20, accompanied by persistent phosphorylation of AKT and PRAS40 (Fig 3A and 3B). We then compared RCC4S and RCC4B20 cells with RCC4B20-S cells that had regained sensitivity to BEZ235 (Fig 2A). The RCC4B20-S cells manifest lower basal levels of phospho-AKT, and the phosphorylation of AKT and 4E-BP1 was fully suppressed by BEZ235, equivalent to the pattern in RCC4S cells (Fig 3C). Thus, these phosphorylation events tracked with BEZ235 resistance in this acquired resistance model. This finding, together with our data (Fig 1A and 1B) and reports of others [22, 23] indicating that growth inhibition tracks with 4E-BP1 phosphorylation, led us to hypothesise that the induced resistance was driven by BEZ235-refractory phosphorylation of 4E-BP1. We also noted that 4E-BP1 phosphorylation has been shown to correlate with primary resistance to mTOR inhibition in a variety of cancer cell lines [39, 40]. It is known that 4E-BP1 sequesters the initiation of translation factor 4E (eIF4E), suppressing cap-dependent translation. When 4E-BP1 is phosphorylated, eIF4E is released, and cap dependent translation can proceed [41]. The activity of eIF4E is also influenced by the ratio of 4E-BP1 and eIF4E [42]. Both eIF4E and 4E-BP1 and their relative abundance have been linked to tumour progression and mTOR kinase resistance [41, 43, 44]. However, we found no evidence for altered expression of eIF4E in BEZ235-resistant RCC4 cells, and neither was there any change in expression of two 4E-BP1 phosphatases: metal-dependent protein phosphatase 1G (PPM 1G) and serine/threonine protein phosphatase 2A (PP2A) [45, 46] (Figure D a-b in S1 File).

To investigate whether altered expression of mTOR complex components could account for increased 4E-BP1 phosphorylation seen in RCC4B20 cells, we assessed levels of RAPTOR and RICTOR, the unique mTOR binding partners in mTORC1 and mTORC2 respectively [2]. This revealed that RCC4B20 cells expressed higher levels of RAPTOR than BEZ235-sensitive RCC4S cells, while levels of RICTOR were unchanged (Fig 3D and 3E). There was also no change in expression of the inhibitory mTORC1 component proline-rich AKT substrate 40 kDa (PRAS40, Fig 3A) [47]. *RPTOR* mRNA was expressed at higher levels in BEZ235-resistant RCC4B20 cells compared with RCC4S controls (130 ± 5%, p<0.001), confirming that RAPTOR was up-regulated at the transcriptional level (Fig 3F). This raised the possibility that changes in the mTORC1 complex contributed to BEZ235-resistant 4E-BP1 phosphorylation in RCC4B20 cells, despite the demonstrable ability of BEZ235 to block mTORC1 activity towards the alternative substrate S6.

### BEZ235 resistance is suppressed by RAPTOR depletion or mTORC1 inhibition

**Fig 4:**
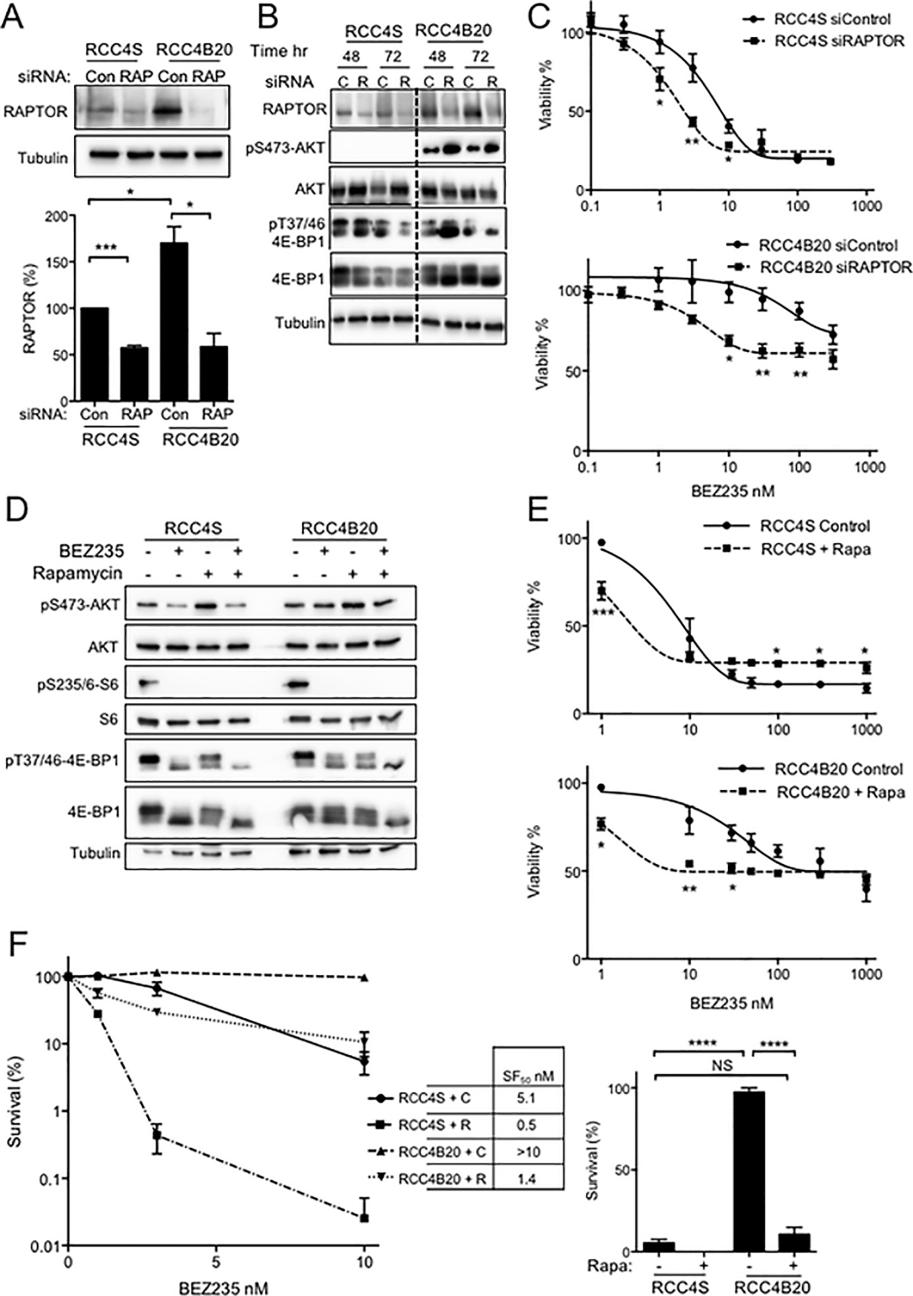
Resistance to BEZ235 is suppressed by targeting mTORC1. **A**. RCC cells were transfected with AllStars control (Con) or RAPTOR (RAP) siRNA, collected after 48 hr and analysed by western blot. Graph below: RAPTOR levels were quantified (n = 4 independent blots) using ImageJ, corrected for tubulin loading and expressed as mean ± SEM % RAPTOR protein in control-transfected RCC4S cells. **B.** RCC4S and RCC4B20 cells were siRNA transfected as A with control (C) or RAPTOR (R) siRNAs. RCC4B20 transfection was conducted in the absence of BEZ235; after 6 hr incubation with siRNA, medium exchange was performed as per protocol, adding solvent control medium to RCC4S cells and 20 nM BEZ235 to RCC4B20 cells to assess effects of RAPTOR depletion on BEZ235-associated p4E-BP1 phosphorylation. Cells were lysed at the indicated times and analysed by western blot. Dotted line: removal of intervening lanes. **C**. Effects of BEZ235 on viability of RAPTOR depleted cells. Upper, RCC4S; lower, RCC4B20 cells were transfected with control or RAPTOR siRNA; after 48 hr to allow RAPTOR depletion, BEZ235 was added and viability assayed 72 hr later. Graphs: mean ± SEM of 4 independent experiments, showing shift of dose-response curve in RAPTOR depleted cells (*p<0.05, **p<0.01; see Table 3 for GI_50_ values). **D.** Cells were treated with solvent, 20 nM BEZ235, 1 nM rapamycin or the combination, collected after 6 hr and lysates were analysed by western blotting. **E.** Relative viability of: upper, RCC4S; lower, RCC4B20 cells treated with BEZ235 alone or with rapamycin (Rapa) as D (*p,0.05, **p<0.01, ***p<0.001; see Table 3 for BEZ235 GI_50_ values). **F.** Relative survival of cells treated as E with BEZ235 with solvent control (C) or rapamycin (R), showing SF_50_ values. Graph to right: relative survival at 10 nM BEZ235 (****p<0.0001). Survival of rapamycin-treated RCC4B20 was not significantly different from survival of RCC4S cells.

We used two approaches to test the contribution of mTORC1 in driving BEZ235 resistance, firstly targeting mTORC1 using RAPTOR siRNA. After 48–72 hr from siRNA transfection, there was detectable RAPTOR depletion, associated with reduction in phospho-4E-BP1 that was most evident in the resistant RCC4B20 cells compared with RCC4S controls (Fig 4A and 4B). We noted increased phosphorylation of S473 AKT in RAPTOR-deleted RCC4B20 cells, suggesting that RAPTOR knockdown did not inhibit mTORC2 function. The increase in phospho-AKT may have resulted from relief of negative feedback between S6K and AKT, consequent upon mTORC1 inhibition [35, 36]. Control-transfected and RAPTOR depleted cells were tested in viability assays to assess effects on basal growth and sensitivity to BEZ235. RAPTOR depletion inhibited cell growth in both RCC4S and RCC4B20 cells, and the effect was similar to that induced by rapamycin (Figure D panel c in S1 File). We then tested effects on response to treatment with BEZ235. In RCC4S cells RAPTOR depletion enhanced sensitivity to BEZ235, with a 2-fold change in BEZ235 GI_50_. RCC4B20 cells showed a more marked shift in the dose-response curve, with a 20-fold reduction in BEZ235 GI_50_ (Fig 4C, Table 3), although there was no change in the response to >100nM BEZ235.

**Table 3:** Effects of mTORC1 targeting on BEZ235 resistance.

Intervention	BEZ235 GI_50_ nM (95% CI)		Fold sensitisation
**RAPTOR depletion**	siControl	siRAPTOR	
RCC4S	4 (3–5)	2 (1–3)	2
RCC4B20	355 (190 - >1000)	18 (8 - >1000)	20
**mTORC1 inhibition**	Control	Rapamycin	
RCC4S	8 (7–10)	2 (2–3)	4
RCC4B20	225 (81 - >1000)	8 (4 - >1000)	28

Cells were transfected with AllStars non-silencing control siRNA (siControl) or siRAPTOR, or treated with solvent or Rapamycin, in the absence or presence of BEZ235. GI_50_ values were interpolated from 3 independent viability assays each with triplicate data points, and Table shows mean GI_50_ (95% confidence intervals). Fold sensitisation calculated as ratio of mean GI_50_ value in solvent-treated or control-transfected cells/GI_50_ in inhibitor-treated or RAPTOR-depleted cells.

Secondly, we targeted mTORC1 using allosteric mTORC1 inhibitor rapamycin, initially comparing the ability of rapamycin and BEZ235 to block 4E-BP1 phosphorylation in parental and BEZ235-resistant cells. Both drugs caused complete inhibition of S6 phosphorylation in RCC4S and RCC4B20 cells. Acute treatment with BEZ235 caused greater inhibition of 4E-BP1 phosphorylation in RCC4S than RCC4B20 cells, while rapamycin alone did not suppress phospho-4E-BP1 in either subline. In contrast, the combination of rapamycin and BEZ235 caused clear suppression of 4E-BP1 phosphorylation in both the sensitive and resistant cells (Fig 4D). These results phenocopied effects of RAPTOR knockdown (Fig 4B), and suggest that mTORC1 was the principal kinase responsible for BEZ235-refractory 4E-BP1 phosphorylation in BEZ235-resistant RCC cells. Finally, we tested effects of BEZ235 alone or with rapamycin on cell viability and survival. In RCC4S cells, rapamycin sensitized to BEZ235 only at 1 nM, with 4-fold change in GI_50_ (Fig 4E upper panel). BEZ235-resistant RCC4B20 cells were sensitised more markedly, with a 28-fold reduction in GI_50_ (Fig 4E lower panel, Table 3). As in RAPTOR depleted cells (Fig 4C) the sensitization effect was apparent at lower (<100 nM) BEZ235 concentrations. Effects of rapamycin were also tested in cell survival assays, with evidence of enhanced BEZ235 response in both RCC4S and RCC4B20 cells (Fig 4F). Of note, in the presence of 10 nM BEZ235, the survival of rapamycin-treated RCC4B20 cells was not significantly different from survival of the sensitive RCC4S subline (Fig 4F, right panel).

## Discussion

Our initial evaluation of mTOR inhibition in RCC cells showed that rapamycin but not BEZ235 enhanced S473 AKT phosphorylation, likely due to relief by rapamycin of negative feedback between S6K and IRS-1, as we reported previously in RCC cells [36]. We noted that the ability of both agents to inhibit proliferation and cell survival tracked with suppression of 4E-BP1 phosphorylation, consistent with reports of other groups [22, 23].

The principal objective of this study was to investigate mediators of resistance to mTOR kinase inhibition, aiming to identify predictive biomarkers and guide selection of drug combinations to combat acquired resistance to mTOR inhibition. We used the approach of exposing RCC cells to dual PI3K-mTOR inhibitor BEZ235. The maximum BEZ235 concentration to which RCC4-EV cells could be adapted was 20nM, lower than the reported Cmax values that range from 22–370 ng/ml (47–788 nM) following administration of BEZ235 200–400 twice daily [48]. None-the-less, such *in vitro* approaches are capable of generating models with clinically-relevant changes, such as the EGFR T790M mutation in gefitinib-resistant non-small cell lung cancer (NSCLC) [26]. Our first major finding was that RCC cells can be induced to be resistant to dual PI3K-mTOR inhibitor BEZ235, and resistant cells are cross-resistant to AZD2014. Despite promising preclinical data, BEZ235 has not shown clinical activity, and proved to have significant toxicity in patients with renal cancer and everolimus-resistant pancreatic neuroendocrine tumors [16, 49]. AZD2014 is tolerable clinically, and in paired tumor biopsies was shown to inhibit S6 phosphorylation [13], although a recent Phase 2 trial in metastatic VEGF-inhibitor refractory RCC was halted early when AZD2014 was found to be inferior to everolimus [50]. However, this agent and other drugs of the same class are the subject of on-going clinical trials, for which predictive biomarkers are required. As an initial step to investigate general mechanisms of BEZ235 resistance, we found that resistant cells showed reversal of resistance on drug withdrawal. Supporting the influence of regulation at the epigenetic level, the resistance phenotype was partially reversed by pan-HDAC inhibitor panobinostat. Previous studies of combined mTORC and HDAC inhibition have highlighted the importance of histone H3 acetylation for maintaining rapalogue sensitivity in RCC cells, via regulation of CDK2/cyclin A [51, 52]. Similarly, combined mTOR and HDAC inhibition were reported to exert synergistic anti-tumor activity in models of breast and pancreatic cancer and NSCLC [53–55]. In a small Phase 1 study, HDAC inhibitor vorinostat with the rapalogue ridaforolimus showed evidence of activity in papillary RCC [56].

Secondly, we found that BEZ235 resistant RCC cells overexpressed and/or activated multiple proteins and pathways, including MET, ABL and MEK-ERK. MET is known to mediate resistance to various cancer therapies, and its over-expression in RCC associates with poor prognosis [57, 58]. ABL is over-expressed in CCRCC [31, 59], although the ABL kinase inhibitor imatinib showed no useful clinical activity in this tumor type [60, 61]. In renal cancer cells including RCC4, 24hr treatment with rapamycin or BEZ235 was shown to activate MEK-ERK signaling, and MEK inhibition sensitised to low nM though not 1 μM BEZ235 [62]. MEK Inhibition was also reported to sensitize to PI3K/mTOR inhibition in head and neck squamous cell cancer [63]. Although not identified as antibody array hits, BEZ235-resistant cells showed dysregulation of two additional pathways, IGF-1R/INSR and Notch. The latter is reportedly up-regulated and activated by PI3K-mTOR inhibition in breast and pancreatic cancer, with data supporting efficacy of combined inhibition of PI3K-mTOR and Notch [64, 65]. However in our model, BEZ235 resistance was not modified by blockade of any of these pathways, although we acknowledge that the simple assays used here may not reflect the true complexity of signalling *in vivo*.

Finally, BEZ235-resistant RCC4B20 cells were shown to display BEZ235-refractory 4E-BP1 phosphorylation, while retaining evidence of phospho-S6 inhibition. It is possible that this discrepancy could highlight intrinsic differences in the response to mTOR inhibition, and indeed we note that both rapamycin and PP242 have been reported to differentially regulate 4E-BP1 and S6K, causing potent inhibition of S6K while 4E-BP1 escape allows maintenance of cap-dependent translation [14, 66]. The persistent 4E-BP1 phosphorylation we detected in BEZ235-resistant RCC4B20 cells appeared to be mTORC1-dependent and driven by RAPTOR up-regulation. In seeking potential explanations for this change in RAPTOR expression, we note that few studies have addressed the regulation of *RPTOR* expression at the transcriptional level. The *RPTOR* promoter contains multiple transcription factor motifs (http://www.genecards.org/cgi-bin/carddisp.pl?gene=RPTOR) and *RPTOR* expression is reportedly regulated via miRNA binding to the 3-UTR of RPTOR mRNA in erythroid cells [67]. In contrast, the regulation of RAPTOR phosphorylation and its interaction with mTOR kinase have been intensively studied [7]. We did not assess RAPTOR post-translational modifications or interactions, and it is possible that these processes could influence expression and/or protein stability. Indeed, we detected greater upregulation of RAPTOR protein in BEZ235-resistance RCCB20 cells than was detected at the mRNA level (Fig 3E and 3F). However, the molecular basis of *RPTOR* mRNA upregulation in BEZ235-resistant RCC4B20 cells is unclear.

Consistent with the concept of RAPTOR as a resistance mediator, BEZ235 resistance and 4E-BP1 phosphorylation were significantly attenuated by targeting mTORC1 via RAPTOR knockdown or rapamycin. Given the association between viability and 4E-BP1 phosphorylation we found earlier (Fig 1) and also reported by others [22, 23], it is plausible that greater suppression of 4E-BP1 phosphorylation (Fig 4D) could account, at least in part, for the increased effect of the combination of BEZ235 and rapamycin. Whilst there are reports that resistance to mTORC kinase inhibition can be associated with altered eIF4E:4E-BP1 ratio [42, 44], there are no reports that increased RAPTOR expression is specifically associated with this resistance phenotype. There are however data supporting a link with adverse outcomes in CCRCC: RAPTOR up-regulation was associated with higher stage and grade, and was a component of an mTOR biomarker panel associated with poor prognosis [68]. RAPTOR acts as a scaffold that is absolutely required for the activity of mTOR, by binding directly to its substrates 4E-BP1 and p70S6 kinase and promoting close apposition with mTOR [69, 70]. Rapamycin is known to inhibit the activity of mTOR when bound to FKBP12, by causing mTOR to dissociate from RAPTOR [9], and RAPTOR depletion or knockout were reported to sensitize HEK293 cells and mouse embryonic fibroblasts to rapamycin-induced retardation of translation [71]. Of clear relevance to our data, *in vitro*-induced everolimus resistance in Caki-1 RCC cells was accompanied by increased pS792 RAPTOR and pT1135 RICTOR phosphorylation (although total levels of these proteins were not assessed), and RAPTOR phosphorylation was suppressed in parental but not everolimus-resistant cells by the natural compound sulforaphane [72]. In addition, everolimus-adapted breast cancer cells were shown to exhibit activation of a lung-metastasis signature and recovery of mTORC1 signaling including RAPTOR upregulation, which was suppressed by depletion of the ecotropic viral integration site-1 proto-oncogene that also reduced *in vitro* and *in vivo* growth and metastasis [73]. RAPTOR dysregulation was also implicated in a study of colorectal cancer: compared with cells that were either mutant or wild-type for both KRAS and PIK3CA, non-isogenic KRAS-mutant cells were reported to be relatively resistant to mTOR kinase inhibitor PP242, and to modestly overexpress RAPTOR [14]. However neither of these two latter studies addressed the specific contribution of RAPTOR to the resistance phenotype [14, 73]. Also relevant to our findings, RICTOR amplification associated with sensitivity to AZD2014 in patients and cell line models of gastric cancer and SCLC [74, 75]. These data are consistent with the results of our study, supporting the hypothesis that increasing the ratio of mTORC1 to mTORC2 activity confers resistance to mTOR kinase inhibition. Further reinforcing this concept, recent data have identified an activating mutation in the mTOR kinase domain as a mediator of *in vitro* induced resistance to AZD8055 [76].

Our data suggest that there may be merit in combining mTOR kinase inhibitors with rapalogues. Indeed, BEZ235 was found to synergize with everolimus in a preclinical model of hepatocellular carcinoma [77]. However, this has proved challenging given the toxicities of BEZ235, which include mucositis, hyperglycemia, dehydration, fatigue and thrombocytopenia [48]. Some of these, including mucositis and hyperglycemia, are in common with other inhibitors of the PI3K-mTOR pathway, and are likely to be class effects [78]. A recent Phase 1b trial reported that BEZ2325 increased everolimus steady state pharmacokinetics, the combination had no objective clinical activity, and even though everolimus was dosed at 2.5 mg daily (below the standard dose of 10 mg), the combination was poorly tolerated with toxicities including fatigue, anorexia, nausea, diarrhea, mucositis, anemia and elevated liver enzymes [79]. The outcomes from this trial suggest that it will be necessary to identify agents with improved tolerability profiles, in order to evaluate the combination of mTOR kinase inhibitor with rapalogue. This may be feasible, given that a number of mTOR inhibitors remain under active clinical investigation. Our identification of RAPTOR up-regulation as a novel mediator of resistance to mTOR kinase inhibition in renal cancer suggests that RAPTOR expression should be included in the pharmacodynamic evaluation of mTOR kinase inhibitor trials.

## Materials and methods

### Cell lines and reagents

RCC4-EV cells that harbor inactivating VHL mutation [21] were obtained from Cancer Research UK Laboratories, Clare Hall, Hertfordshire, UK. Cells were cultured in DMEM medium with 10% fetal calf serum (FCS) with 1% penicillin/streptomycin (Gibco) and 0.5mg/ml G418 (Invitrogen). Cells were tested every ~3 months with MycoAlert (Lonza Rockland Inc.), and were mycoplasma-free. BEZ235-resistant RCC4 cells were generated by continuous culture in BEZ235, increasing the concentration as allowed by recovery of proliferation rate, as in [26, 80]. Control cultures were treated with medium containing 0.1% DMSO. Media were exchanged for fresh solvent-containing or BEZ235-containing media at least every 7 days. Parental (BEZ235-sensitive) and BEZ235-resistant RCC4 and were authenticated as RCC4 by STR genotyping, with no differences between parental and resistant sublines (Eurofins Medigenomix Forensik GmbH).

Rapamycin (5 mM solution in DMSO, Calbiochem) was aliquoted and stored at -20°C, protected from light. Other inhibitors were supplied as powder and were dissolved in DMSO and stored at -20°C or -80°C. The following agents were from Selleckchem: BEZ235, BKM120 (PI3K inhibitor), AZD2014 (mTOR kinase inhibitor), OSI-906 (IGF-1R/INSR inhibitor), AZD5363 (AKT inhibitor), AZD6244 (selumetinib, MEK inhibitor), panobinostat (HDAC inhibitor), INC280 (MET inhibitor), imatinib (ABL kinase inhibitor). To treat cells, OSI-906 was diluted in DMSO and further diluted in pre-warmed complete medium containing 5% DMSO to produce a 10X stock solution, as described [81]. Other agents were diluted to the required concentration in complete medium with 10% FCS. Control cells were treated with complete medium containing an equivalent volume (0.1%) of DMSO. To induce gene silencing, cells were reverse transfected with siRNA using DharmaFECT1 reagent (Dharmacon, USA) according to the manufacturer’s protocol, using siRNAs to deplete MET (H_MET_10, Qiagen) or RAPTOR (SASI_HS01_00048380, Sigma-Aldrich), or AllStars non-silencing control siRNA (Qiagen).

### Assays for viability, cell survival, apoptosis

After a 72 hr drug-free washout period, viability was quantified by CellTiter Glo assay (Promega) and cell survival by clonogenic assay, as described [81]. Apoptosis was measured using Caspase-Glo 3/7 assay (Promega) according to the manufacturer’s protocol, luminescence was read on a Fluorstar Optima luminometer (BMG Labtech), and expressed as % signal in solvent-treated controls.

### Antibody array, immunoprecipitation, western blotting and qRT-PCR

The Phospho Explorer Array (www.fullmoonbiosystems.com) was used to screen protein expression and phosphorylation, using 1318 proprietary antibodies printed onto glass slides. Parental (drug-sensitive) RCC4 cells were treated with 20 nM BEZ235, or solvent control (0.1% DMSO), and BEZ235-resistant RCC4 cells underwent medium exchange for fresh medium containing 20nM BEZ235. After one hour, cells were collected, cell pellets were snap frozen and transferred on dry ice to Full Moon Biosystems (Sunnyvale, CA) for antibody array analysis. Normalised data were filtered to exclude antibodies that did not generate average signal intensity in replicate spots of at least 3 times the background signal. Immunoprecipitation and western blot were performed as in [81, 82], using antibodies listed in Table A in S1 File. Results shown are representative of 2–5 independent experiments. To quantify expression at the mRNA level, total RNA was extracted using the RNeasy micro kit (Qiagen, UK), and reverse transcribed using the SuperScript III First-Strand Synthesis System for RT-PCR (Invitrogen, Life Technologies). cDNAs were amplified using primers listed in Table B in S1 File and Sybr Green PCR Mix (Applied Biosystems) on a 7500 Fast RT-PCR System (Applied Biosystems).

### Statistical analysis

Data were analysed and graphed using Excel (Microsoft, USA) and Prism v6 (GraphPad, USA). Graphs show mean and standard error of mean (SEM). Student’s t-test was used to compare two groups of data, one-way analysis of variance (ANOVA) to compare more than two groups of data, and two-way ANOVA to compare datasets including two variables. Non-linear regression was used to calculate GI_50_ and SF_50_ values, the concentrations of drug required respectively to inhibit growth and cell survival to 50% of control levels.

## Supporting information

S1 File **Table A**: Antibodies used for western blotting and immunoprecipitation.
**Table B:** Sequences of primers used for qPCR.
**Figure A.** Testing response of RCC cells to PI3K-AKT-mTOR and HDAC inhibition.
**Figure B.** Multiple signalling pathways are activated in BEZ235-resistant RCC cells.
**Figure C.** BEZ235 resistance is not reversed by targeting MET, ABL, MEK, IGF-1R or Notch.
**Figure D.** BEZ235-resistant RCC4 cells do not show altered expression of eIF4E or 4E-BP1 phosphatases, and respond similarly to RAPTOR depletion and rapamycin.
(PDF)

S2 File **Table A:** Antibody array data.
**Table B:** Signal ratio of phosphoprotein to non-phosphoprotein.
(XLSX)
