# Supplementary material for: RAPTOR up-regulation contributes to resistance of renal cancer cells to PI3K-mTOR inhibition
Source: PLoS One. 2018 Feb 1;13(2):e0191890. doi: 10.1371/journal.pone.0191890 (PMC5794101; doi:10.1371/journal.pone.0191890)
Supplement: S1 File — Table A: Antibodies used for western blotting and immunoprecipitation. Table B: Sequences of primers used for qPCR. Figure A. Testing response of RCC cells to PI3K-AKT-mTOR and HDAC inhibition. Figure B. Multiple signalling pathways are activated in BEZ235-resistant RCC cells. Figure C. BEZ235 resistance is not reversed by targeting MET, ABL, MEK, IGF-1R or Notch. Figure D. BEZ235-resistant RCC4 cells do not show altered expression of eIF4E or 4E-BP1 phosphatases, and respond similarly to RAPTOR depletion and rapamycin. (PDF) [file pone.0191890.s001.pdf]

S1 File: RAPTOR up-regulation contributes to resistance of renal cancer cells to PI3K-mTOR inhibition.

Philip Earwaker, Caroline Anderson, Frances Willenbrock, Adrian L. Harris, Andrew S. Protheroe, Valentine M. Macaulay.

S1 File contains:

Table A in S1 file: Antibodies used for western blotting and immunoprecipitation

Table B in S1 file: Sequences of primers used for qPCR

Figure A in S1 file. Testing response of RCC cells to PI3K-AKT-mTOR and HDAC inhibition.

Figure B in S1 file. Multiple signalling pathways are activated in BEZ235-resistant RCC cells.

Figure C in S1 file. BEZ235 resistance is not reversed by targeting MET, ABL, MEK, IGF-1R or Notch.

Figure D in S1 file. BEZ235-resistant RCC4 cells do not show altered expression of eIF4E or 4E-BP1 phosphatases, and respond similarly to RAPTOR depletion and rapamycin.

Antibody	Source (Catalog number)
4E-BP1	Cell Signaling (#9644)
c-ABL	Cell Signaling (#2862)
Acetyl-Histone H3	Merk Millipore (#06-599)
β-Actin	Abcam (ab8224)
AKT	Cell Signaling (#9272)
CRK II	Cell Signaling (#3492)
eIF4E	Cell Signaling (#2067)
HES1	Gift of Dr T. Sudo, Toray Industries Japan *
IGF-1R β	Cell Signaling (#3027)
INSR β	Cell Signaling (#3025)
Jagged 1	Cell Signaling (#2620)
MEK 1/2	Cell Signaling (#4694)
MET	Cell Signaling (#8198)
Notch1 (Cleaved) (Val1744)	Cell Signaling (#4147)
P44/42 MAPK (Erk1/2)	Cell Signaling (#4695)
PARP	Cell Signaling (#9542)
Phospho-4E-BP1 (Thr37/46)	Cell Signaling (#2855)
Phospho-AKT (Ser473)	Cell Signaling (#4051)
Phospho-CRKII (Tyr221)	Cell Signaling (#3491)
Phospho-IGF-1R β (Tyr1135/6 / INSR β Tyr1150/1)	Cell Signaling (#3024)
Phospho-MEK1 (Ser221)	Cell Signaling (#2388)
Phospho-MET (Tyr1234/1235)	Cell Signaling (#3077)
Phospho-MET (Tyr1356)	Abnova (#PAB0536)
Phospho-MET (Tyr1356)	Abgent (#AP3168a)
Phospho-p44/42 MAPK (Erk1/2) (Thr202/Tyr204)	Cell Signaling (#4377)
Phospho-PRAS40 (Thr246)	Cell Signaling (#2640)
Phospho-S6 Ribosomal Protein (Ser235/236)	Cell Signaling (#2211)
PP2A C	Cell Signaling (#2259)
PPM1G	Bethyl Laboratories (#A300-880A)
PRAS40	Cell Signaling (#2610)
RAPTOR	Cell Signaling (#2280)
RICTOR	Cell Signaling (#9476)
S6	Cell Signaling (#2217)
β-Tubulin	Sigma-Aldrich (#T4026)

Table A in S1 file. Antibodies used for western blotting and immunoprecipitation. *HES1 antibody was a gift of Dr Sudo, as described in Aleksic T, Feller SM. Gamma-secretase inhibition combined with platinum compounds enhances cell death in a large subset of colorectal cancer cells. Cell Communication and Signaling, 2008;6:8. Epub 2008/10/28. doi: 10.1186/1478-811x-6-8.

Gene	RefSeq	Forward Primer	Reverse Primer
HEY1	NM_001040708	CGAGCTGGACGAGCCCAT	GGAACCTAGAGCCGAACTCA
HES1	NM_005524	CAGCCAGTGTCAACACGACA	GTGCGCACCTCGGTATTAAC
JAG1	NM_000214	CCAGTGTCAGAATGACGCCT	ACTCTTGCACTTCCCGTGAG
RPTOR	NM_001163034	TCTGTCGGCATCTTCCCCTA	CCAGCTCGCTGTCCACTG
ACTB	NM_001101	CCCAGCACAATGAAGATCAA	CGATCCACACGGAGTACTTG

Table B in S1 file. Sequences of primers used for qPCR. All sequences are 5’ to 3’.

Figure A in S1 File

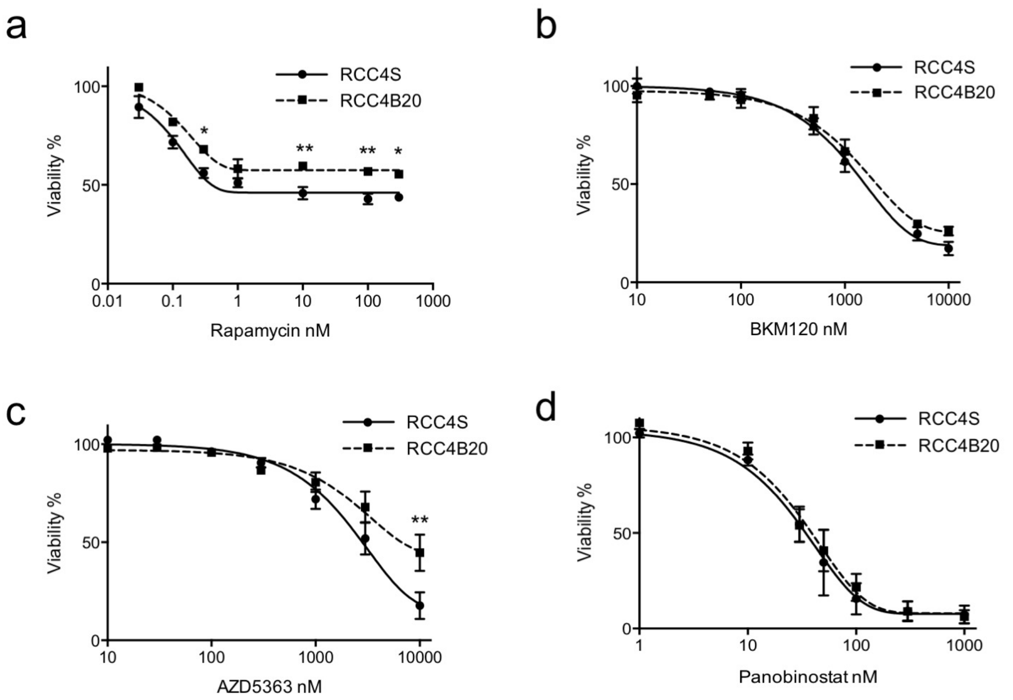

Figure A in S1 file. Testing response of RCC cells to PI3K-AKT-mTOR and HDAC inhibition. Cells were treated with **a**, rapamycin; **b**, BKM120; **c**, AZD5363, and relative viability assayed after 72 hr. Graphs show mean ± SEM of 3 independent experiments. BEZ235-resistant RCC4B20 cells showed no or minor cross-resistance to these agents (*p<0.05, **p<0.01). **d.** Cells were treated with Panobinostat and viability assayed as a-c above. Panobinostat caused comparable dose-dependent growth inhibition of RCC4S and RCC4B20 cells.

Figure B in S1 File

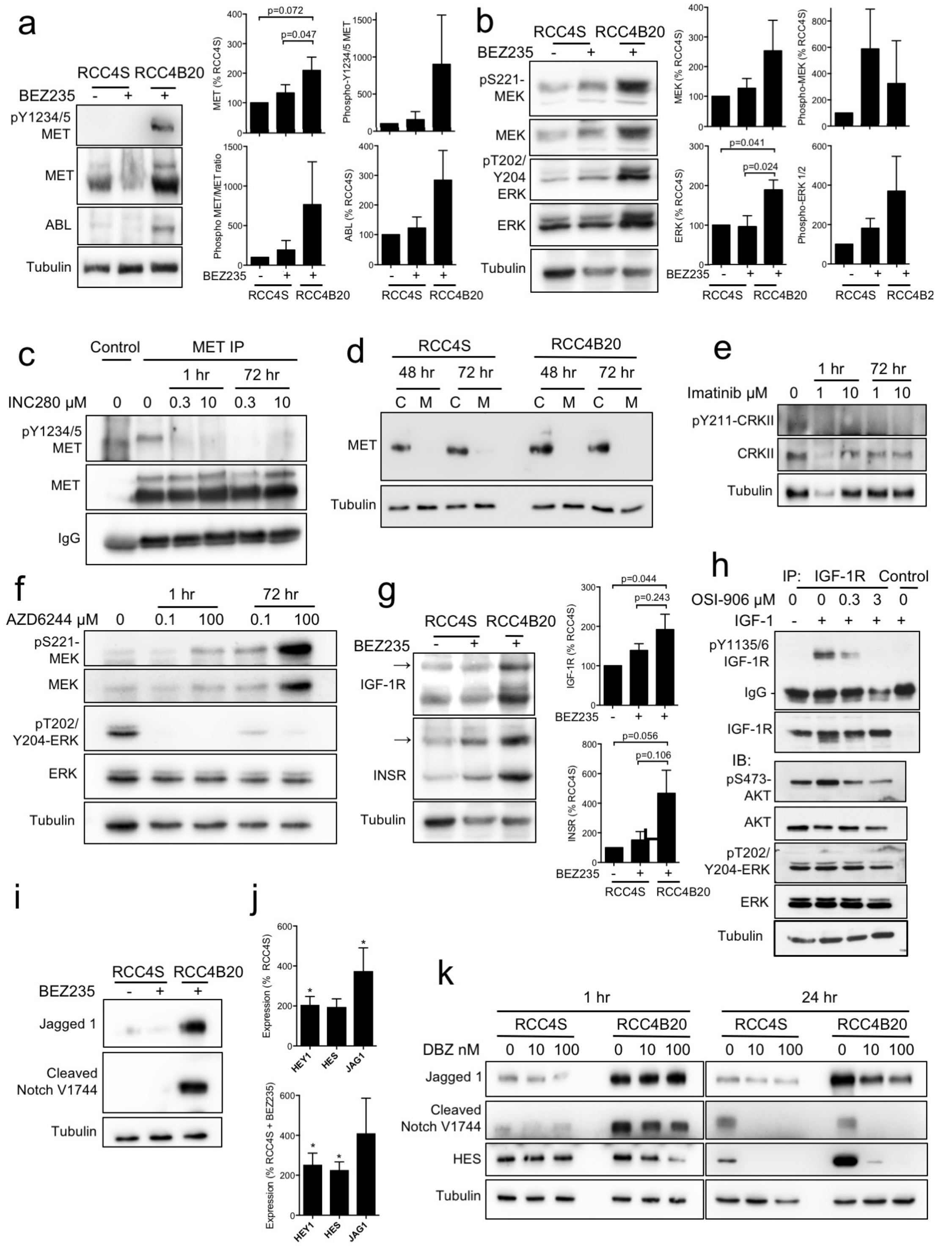

Figure B in S1 file. Multiple signalling pathways are activated in BEZ235-resistant RCC cells. a, b. RCC4S cells were treated with solvent or BEZ235 for 1 hour, and resistant RCC4B20 cells underwent medium exchange for fresh medium containing 20 nM BEZ235, to control for effects of replenishing new medium. Cell lysates were analysed by western blotting using the indicated antibodies. Graphs to right show quantification of replicate (n=3) blots, normalized for loading. **c.** RCC4B20 cells were treated with solvent or MET inhibitor INC280 for the indicated times, whole cell extracts were immunoprecipitated with MET or control IgG, and analyzed by western blotting for phospho- and total MET. **d**. RCC cells were reverse transfected with MET siRNA (M) or non-silencing Allstars control siRNA (C). Cells were lysed at the indicated times and analysed

by western blot. Similar results were seen in a second independently prepared set of cell lysates. **e.** Cells were treated with solvent or Imatinib for 1 or 72 hr and analyzed by western blotting for phosphorylated and total CRKII to test for ABL inhibition. **f.** RCC4S cells were treated with solvent or AZD6244 and lysates analysed by western blotting for total and phosphorylated MEK and ERK. **g.** Cells were treated as a) and analyzed by western blotting for IGF-1R and INSR. Arrows: IGF-1R and INSR proreceptors. Graphs to right: replicate blots (5 for IGF-1R, 4 for INSR) were quantified, normalised for loading and expressed as % control-treated RCC4S cells. **h.** Cells were serum-starved overnight and treated with OSI-906 for one hour and in the final 15 min with 50 nM long R3-IGF1 (Sigma-Aldrich). Whole cell lysates were immunoprecipitated with IGF-1R or control antibody and immunoblotted for total and phosphorylated IGF-1R, showing that IGF-1R activation was inhibited partially by 0.3 μM and completely by 3 μM OSI-906. Parallel lysates were immunoblotted (IB) for total and phosphorylated AKT and ERKs. **i, j.** Cells were treated as a) and: **i**, analyzed by western blotting for Jagged 1 and cleaved Notch 1 V1744 to detect NICD; **j**, used for RNA extraction and qRT-PCR. Graphs: gene expression in RCC4B20 cells (mean ± SEM of 4 independent experiments) normalised to actin and expressed relative to: upper, solvent-treated RCC4S cells; lower, BEZ235-treated RCC4S cells (*p<0.05). **k.** After 3 day drug washout, cells were treated with solvent or gamma secretase inhibitor DBZ for 1 or 24 hr and analyzed by western blotting for Jagged, cleaved Notch 1 V1744 (NICD) and HES as a downstream Notch regulated gene.

Figure C in S1 File

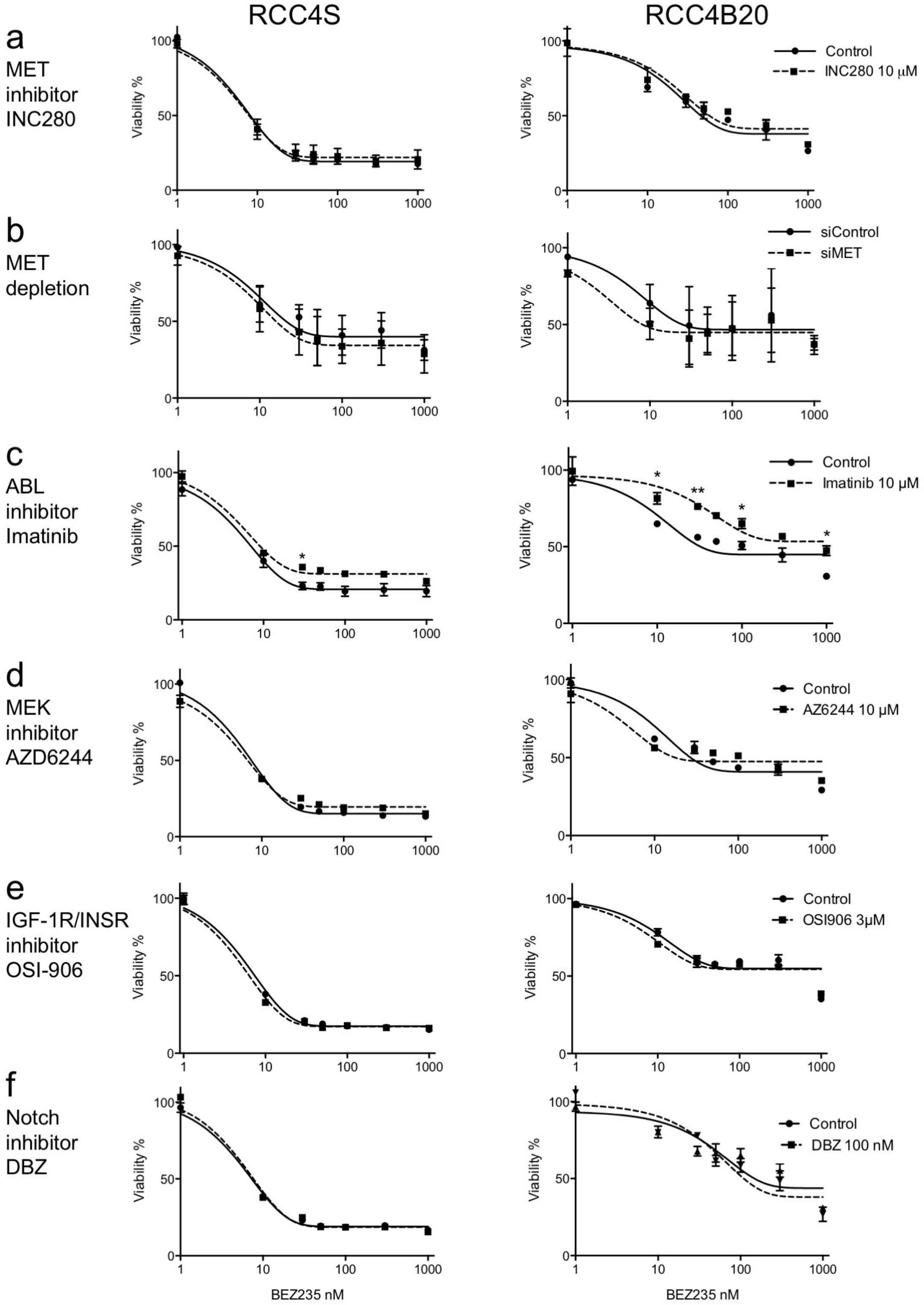

Figure C in S1 file. BEZ235 resistance is not reversed by targeting MET, ABL, MEK, IGF-1R or Notch. a. RCC4S (left) and RCC4B20 (right) were treated with BEZ235 alone or with 10 μM INC280. Graphs show relative viability expressed as mean ± SEM of two independent experiments, each with triplicate data points. **b.** RCC4S and RCC4B20 cells were transfected with control siRNA or MET siRNA, 48 hours later treated with BEZ235, and after a further 72 hr

viability was assayed. Graphs: mean and SEM of 6 data points from two independent experiments. MET inhibition or depletion did not influence the response of RCC4S or RCC4B20 cells to BEZ235 ($p>0.05$ by two-way ANOVA). **c-f**. RCC4S (left) and RCC4B20 (right) were treated with BEZ235 alone or with **c,** 10 μM imatinib; **d,** 10 μM AZD6244; **e,** 3 μM OSI-906; **f,** 100 nM DBZ. None of these agents enhanced the response of RCC4B20 cells to BEZ235; indeed imatinib appeared to induce minor increase in resistance (*p,0.05, **$p<0.01$).

Figure D in S1 File

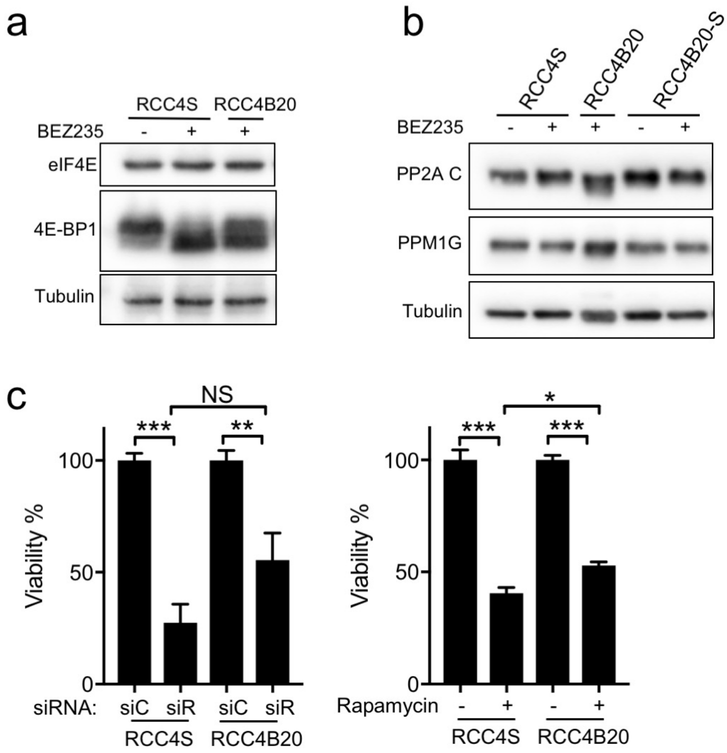

Figure D in S1 file. BEZ235-resistant RCC4 cells do not show altered expression of eIF4E or 4E-BP1 phosphatases, and respond similarly to RAPTOR depletion and rapamycin. a. Cells were treated as Figure B panel a) in S1 File and lysates were western blotted for total eIF4E and 4E-BP1. **b.** Cells were treated as a) and western blotted for 4E-BP1 phosphatases PP2A C and PPM1G. **c.** Cells were: left, transfected with control siRNA (siC) or siRAPTOR (siR); right, treated with solvent control or 1 nM rapamycin. All wells were treated 48hr later with solvent in the absence of BEZ235, and viability was assayed 72hr later. Data are expressed as % viability of the appropriate control (*p,0.05, **p<0.01, ***p<0.001).
